# Regenerative Approach for Improving Flap Survival: Perspective of Angiogenesis

**DOI:** 10.3390/biomimetics11030186

**Published:** 2026-03-04

**Authors:** Se Hyun Yeou, Yoo Seob Shin

**Affiliations:** 1 Department of Otorhinolaryngology-Head and Neck Surgery, Korea University Ansan Hospital, Korea University College of Medicine, Ansan 15355, Republic of Korea; scieth17@ajou.ac.kr; 2 Department of Otorhinolaryngology-Head and Neck Surgery, Ajou University School of Medicine, Suwon 16499, Republic of Korea

**Keywords:** surgical flaps, angiogenesis, microcirculation, regenerative medicine

## Abstract

Flap reconstruction remains a cornerstone after oncologic resection, trauma, and complex wounds, yet partial necrosis, venous congestion, and delayed healing continue to drive morbidity and unplanned re-exploration. Even when macroscopic inflow and outflow are re-established, distal and border-zone tissue may remain constrained by microcirculatory dysfunction. This review frames flap compromise as a biomimetics-relevant failure of a hierarchical transport network and summarizes the vascular repair mechanisms that regenerative interventions aim to replicate. We outline key concepts governing flap perfusion, including angiosomes, choke vessels, endothelial barrier failure, mural cell support, and immune regulation within the angiogenic niche, and relate these to no-reflow, thrombo-inflammation, and impaired vascular regeneration. We then synthesize regenerative strategies aimed at durable reperfusion, spanning recombinant factors, gene and nucleic acid delivery, cell-based therapies, cell-free biologics, including extracellular vesicles and platelet-derived products, pharmacologic modulators, and biomaterial platforms that localize and sustain bioactivity. Translation will require functional perfusion endpoints, standardized reporting of delivery parameters, and safety-conscious designs that minimize aberrant angiogenesis and vector-related risks in post-resection settings.

## 1. Introduction

Reconstructive flap surgery is a fundamental technique in plastic and reconstructive surgery, offering durable coverage and functional restoration for complex defects resulting from oncologic resection, trauma, infection, and chronic wounds. Although advances in microsurgical techniques and perioperative workflows have improved overall success rates, flap compromise remains a significant clinical concern [1,2]. It may manifest as partial necrosis, venous congestion, delayed healing, unplanned re-exploration, or, less commonly, total flap loss, all of which substantially impact patient outcomes and resource utilization [1,3].

Flap compromise is not always attributable to macrovascular patency [4,5]. Even with technically successful arterial and venous anastomoses, distal and watershed territories may fail to re-establish stable microvascular perfusion due to ischemia–reperfusion injury, endothelial barrier dysfunction, leukocyte–platelet activation, and microcirculatory “no-reflow” phenomena [3,4,5,6,7]. This microcirculatory bottleneck results in spatially heterogeneous hypoxia, which can delay healing and increase susceptibility to secondary thrombosis and tissue loss [5,6,7].

Accordingly, vascular growth and stabilization processes—including angiogenesis, arteriogenesis, and remodeling—represent actionable targets to support durable reperfusion [6,8,9,10]. Concurrently, advances in regenerative medicine and biomaterials have enabled biomimetic approaches such as gene-based delivery, extracellular vesicle–based therapeutics, and engineered hydrogels that provide controlled presentation of biological cues [11,12,13]. Biomimetic strategies in this field aim to replicate key features of endogenous vascular repair, including spatiotemporal cue gradients, extracellular matrix–guided niche support, and remodeling programs that ultimately restore hemodynamically competent perfusion. In this review, we synthesize current understanding of flap microcirculatory failure and critically evaluate bioactive, pro-regenerative strategies targeting angiogenesis and vascular remodeling. We then systematize the preclinical evidence and delineate key barriers to clinical application, with emphasis on translational feasibility and safety.

## 2. Vascular Biology and Pathophysiology of Flap Survival

### 2.1. Vascular Architecture and Microcirculation of Flaps

Flap vascular architecture can be understood as a hierarchy of vascular territories and inter-territorial connections that collectively determine microcirculatory stability, particularly at the distal flap, where perfusion reserve is minimal. At the tissue level, flap viability is governed by the integrity of the arteriolar-capillary-venular unit, with capillary recruitment and venous outflow capacity serving as critical determinants [14,15,16,17,18]. Within this framework, the angiosome concept describes three-dimensional tissue blocks supplied by a specific source artery and vein, providing a practical map of expected perfusion and drainage [19,20,21]. Importantly, angiosome borders are not rigid anatomical walls but functional boundaries defined by the caliber and patency of linking vessels, which range from true anastomoses to reduced-caliber choke vessels [20,21,22]. This territorial model is central to safe flap design because it predicts which regions are supported by a robust microvascular network within a territory and which regions must rely on cross-territory recruitment under marginal perfusion conditions.

Within this territorial context, the flap pattern reflects how directly the design captures a dominant vascular axis, thereby supporting capillary perfusion pressure and venous drainage throughout the flap. Axial pattern flaps incorporate a known, named artery and vein along the long axis, providing a higher-capacity, more predictable inflow and outflow pathway that results in more stable distal microcirculation. In contrast, random pattern flaps rely primarily on the subdermal plexus without a defined axial vessel, making microcirculatory perfusion more variable and more sensitive to vasoconstriction, edema, and venous congestion, especially in the distal portion, where cross-territory flow is required [23,24]. Clinically, this distinction explains why axial flaps generally tolerate physiological stress and length extension more reliably than random flaps, even though both remain constrained by territorial boundaries at the microvascular level [23,25].

Flap elevation reduces peripheral microvascular inflow and increases reliance on choke-zone conductance. In this context, distal capillary perfusion and venous outflow become key determinants of flap viability. The distal territory is primarily supplied through choke vessels that connect adjacent angiosomes and serve as physiological resistance points regulating cross-territory flow. The necrosis line often develops at the choke zone, underscoring its role as a flow-limiting boundary [21,26]. After flap elevation and ischemic stress, choke vessels progressively remodel and dilate to function as true anastomoses [21]. Serial angiography and histological studies demonstrate a stepwise increase in choke-vessel caliber over 3–7 days following elevation or surgical delay in animal models [27,28,29]. As boundary resistance decreases, distal capillary recruitment improves, additional angiosome territory is incorporated into the perfused field, and flap survival is enhanced [21,26].

Over longer time scales, flap viability is further stabilized by microvascular integration with the recipient site [30]. Neovascularization from the recipient bed involves the ingrowth of new vessels and the establishment of functional microvascular connections between the wound bed and the flap. This process, often discussed alongside inosculation, describes how capillary networks become continuous [31]. As these connections mature, the flap gradually becomes less dependent on its original pedicle, a transition known as autonomization [32]. The timing and extent of recipient-bed contribution vary depending on flap type and local conditions and are influenced by factors that directly impair microcirculation, such as fibrosis, inflammation, and prior irradiation [33]. Experimental and clinical observations indicate that recipient-derived perfusion can become significant within days to weeks and may be clinically relevant if pedicle compromise occurs after microvascular integration has partially matured [30,33,34]. Taken together, these concepts emphasize that flap survival is ultimately a microcirculatory issue: tissue loss often reflects failure of capillary-level perfusion and venous drainage at territorial borders rather than a simple absence of proximal arterial inflow [35,36,37].

### 2.2. Angiogenesis and Arteriogenesis in Ischemic Flaps

Ischemic flaps restore perfusion through two partially overlapping vascular programs: angiogenesis, which expands the capillary network, and arteriogenesis, which enlarges and remodels pre-existing arteriolar connections into functional collateral conduits. These processes are coordinated across multiple vascular compartments and evolve over time as metabolic demand, shear stress, and inflammatory signaling change within both the flap and the recipient bed [38,39].

Sprouting angiogenesis is the primary early mechanism of microvascular expansion in ischemic tissues, driven by gradients of VEGF family ligands and other hypoxia-responsive signals. Current frameworks recognize that angiogenesis can occur through several modes—such as sprouting, intussusceptive splitting, and coalescent remodeling—but sprouting remains the most well-characterized and consistently observed mode in ischemia-driven repair processes relevant to flap salvage [40,41]. In experimental flap models, the well-known surgical delay phenomenon demonstrates how preconditioning can enhance local pro-angiogenic signaling, including increased VEGF expression, thereby improving subsequent flap survival after transfer or completion of elevation [42].

At the cellular level, sprouting begins with endothelial activation and tip-cell selection, followed by stalk-cell proliferation, lumen formation, and connection to neighboring sprouts to establish perfused circuits. Tip-stalk patterning is stabilized by Notch signaling downstream of VEGF, which limits excessive branching while permitting directional growth toward ischemic gradients [41,43]. Because ischemic flaps often depend on marginal perfusion zones, the balance between productive sprouting and non-productive, leaky neovessels is clinically significant, especially when inflammation and edema increase interstitial pressure and reduce effective microvascular flow [38,41].

Arteriogenesis, the outward remodeling of pre-existing collateral arterioles, differs mechanistically from sprouting angiogenesis and is often initiated by altered hemodynamics rather than hypoxia alone. When blood flow is redirected through pre-existing anastomoses, increased fluid shear stress activates the endothelium, induces adhesion molecules and chemokines, and promotes the recruitment of circulating monocytes that support the outward remodeling of collateral arterioles [39,44]. In flap physiology, this concept is directly relevant to the arteriogenic recruitment of connecting vessels that compensate for reduced inflow, as well as to the maturation of newly established networks into higher-conductance pathways capable of sustaining tissue-level perfusion [38,39].

Hypoxia and the hypoxia-inducible factor (HIF) pathway integrate metabolic stress with pro-angiogenic transcriptional programs across endothelial and stromal cells. Under low oxygen tension, HIF stabilization promotes the expression of VEGF, glycolytic regulators, and multiple ancillary angiogenic mediators that influence endothelial migration, proliferation, and survival [45,46]. The apelin–APJ axis is a representative HIF-responsive pathway with documented roles in endothelial proliferation and regenerative angiogenesis, highlighting that hypoxia-driven repair is not mediated by VEGF alone [47]. Consistent with this, recent studies on human vascular cells demonstrate that intact HIF-1α signaling is essential for robust ischemic vascular regeneration, and that disrupting HIF-dependent programs in specific vascular lineages can impair pro-angiogenic responses [48].

Endothelial activation, pericyte recruitment, and vascular remodeling collectively determine whether early neovessels persist and become functional. As sprouts stabilize, mural cell coverage and extracellular matrix deposition help reduce leakiness, enhance vasomotor competence, and support long-term network integrity [41,49]. Pericyte recruitment is strongly associated with PDGF-B and related signaling pathways, and insufficient mural support is classically linked to fragile microvessels and abnormal remodeling [49,50]. In ischemic flaps, increases in capillary density or endothelial markers do not necessarily result in effective reperfusion, particularly when barrier dysfunction, interstitial edema, and venous outflow limitations impair microvascular conductance. This distinction highlights why sustained perfusion depends on vessel maturation with adequate pericyte coverage, as well as hemodynamic competence and efficient venous drainage [38,41].

Finally, macrophage–endothelium crosstalk provides a time-resolved immune “switch” that shapes both sprouting and remodeling. Ischemic injury activates macrophages and initiates local inflammatory responses; M1-like phenotypes tend to predominate early, amplifying inflammation and clearing debris, whereas M2-like phenotypes accumulate later and support angiogenesis and repair [51]. Importantly, the persistence of excessive M1-like activity into later phases is associated with delayed resolution and aggravated tissue injury, which can result in non-productive angiogenesis and impaired stabilization in ischemic tissues [51,52]. Mechanistically, macrophages promote sprouting by supplying pro-angiogenic mediators such as VEGF family signals and by shaping matrix remodeling environments. They also physically support vascular network assembly by localizing near sprouts and facilitating anastomosis in developmental and repair contexts [52,53]. As repair progresses, macrophages contribute to pruning and regression programs that refine the network, illustrating why the timing and phenotype of macrophage responses can determine whether ischemia-driven neovascularization culminates in a stable, efficient circulation or a disorganized and fragile microvascular bed [26,52].

### 2.3. Pathophysiology of Flap Failure

**Figure 1:**
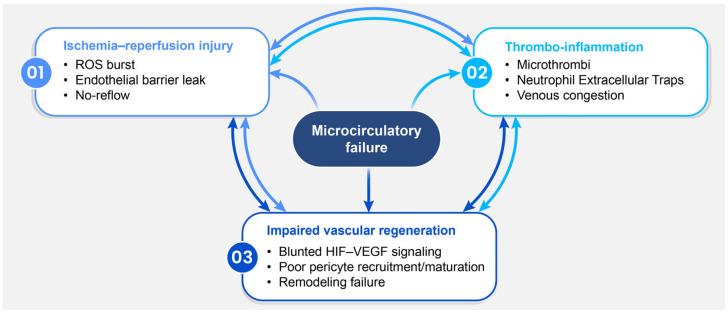
Microcirculatory failure as a convergent mechanism of flap failure.

Flap failure is often clinically characterized as a problem of macroscopic inflow or outflow at the pedicle [54]. However, even when proximal patency appears preserved, the primary determinant of tissue survival is frequently microcirculatory failure within distal and border-zone territories [55,56]. From this perspective, flap failure reflects a convergent collapse of capillary-level perfusion and venous drainage driven by three interconnected mechanisms: ischemia–reperfusion injury involving oxidative stress and regulated cell death; thrombo-inflammation characterized by microthrombosis and maladaptive immune persistence; and impaired angiogenic signaling resulting in defective vascular remodeling (Figure 1).

First, ischemia–reperfusion injury primes the flap microvasculature for dysfunction. During ischemia, ATP depletion, ionic imbalance, and metabolic acidosis compromise endothelial homeostasis. Upon reperfusion, oxygen delivery to enzymatic and mitochondrial systems shifts toward reactive oxygen species (ROS) generation, producing an oxidative burst that amplifies lipid peroxidation, mitochondrial injury, and downstream inflammatory signaling [57,58]. The conversion of xanthine oxidoreductase to xanthine oxidase activity has been identified as a significant source of ROS in flap ischemia–reperfusion contexts [59]. A key microvascular consequence is endothelial barrier disruption, leading to capillary leakage and interstitial edema. Increased interstitial pressure can compress low-pressure venules and capillaries, slow microvascular flow, and contribute to heterogeneous reperfusion with persistent non-perfused regions, consistent with no-reflow patterns that disproportionately affect distal territories [55,57]. In experimental microvascular free flap models, ischemia–reperfusion has been associated with reduced flap survival and histologic evidence of edema with inflammatory infiltration, supporting barrier failure and early tissue injury as structural correlates of impaired perfusion recovery [60]. Pharmacologic data further support the pathogenic role of ROS-linked cascades, as xanthine oxidase inhibition with febuxostat attenuated oxidative stress and inflammatory markers in an animal skin flap ischemia–reperfusion model [61].

Second, thrombo-inflammation exacerbates microvascular obstruction [62]. Oxidative endothelial injury and endothelial activation promote leukocyte adhesion, platelet activation, and engagement of the coagulation pathway, collectively predisposing to microthrombi formation, capillary plugging, and secondary perfusion deficits. These processes are mutually reinforcing, as inflammatory mediators can further increase ROS generation, while barrier leakage and reduced shear stress facilitate leukocyte–platelet interactions [58,63,64]. Neutrophil extracellular trap (NET)-associated mechanisms have been proposed as a link between innate immune activation and microvascular obstruction by providing a scaffold that promotes platelet aggregation and coagulation activation, thereby amplifying microthrombosis [65]. Concurrently, macrophage programs influence whether inflammation resolves and tissue repair ensues or persists in a tissue-injuring state that sustains endothelial activation and impairs perfusion recovery [66]. In a rat microvascular free flap study, macrophage-associated expression markers, including Arginase 1 (ARG1) and the ARG1/Nitric Oxide Synthase 2 (NOS2) ratio, were reported to change after ischemia–reperfusion, consistent with disruption of regulatory programs relevant to inflammatory resolution [60].

Third, durable flap viability requires coordinated regenerative remodeling of the microvasculature. Beyond the acute injury phase, angiogenic sprouting, inosculation with the recipient bed, vessel stabilization, and restoration of barrier integrity are essential to convert transient reperfusion into stable territorial perfusion [67]. Hypoxia-inducible programs and canonical angiogenic pathways—including VEGF signaling and angiopoietin-mediated stabilization—are therefore central to recovery after ischemia–reperfusion [68,69,70]. However, excessive exposure to ROS and persistent inflammatory cytokine pressure can impair endothelial responsiveness, compromise pericyte recruitment and maturation, and bias remodeling toward immature, leaky microvessels that fail to stabilize perfusion [58,71,72]. In the rat microvascular flap model, significant alterations in angiogenesis-related transcripts—including VEGFA, FGF2, and ANGPT2—along with the hypoxia-related gene HIF-1α, were reported following ischemia–reperfusion. These findings support the concept that the injury program can disrupt subsequent regenerative signaling necessary for microvascular recovery [60].

Microcirculatory failure is best understood as a set of interconnected, reinforcing loops rather than as independent processes [73]. ROS-driven endothelial dysfunction and barrier leakage promote edema and heterogeneous reperfusion, which facilitate leukocyte–platelet interactions, NET-associated obstruction, and microthrombosis. Persistent inflammatory activation further suppresses productive angiogenic signaling and vessel maturation, resulting in fragile microvessels that cannot stabilize perfusion [58,65,74]. Notably, therapeutic interventions that improve flap survival often act across multiple axes, frequently converging on immunomodulation combined with attenuation of downstream cell death. This is exemplified by Cu-DHM nanozymes, which have been reported to reduce neutrophil infiltration, modulate immune balance, and inhibit apoptosis, alongside improved flap outcomes [75]. Overall, these mechanisms can sustain territorial hypoperfusion in distal and border-zone regions, resulting in partial or total flap failure despite the restoration of macroscopic inflow [58,73,74].

## 3. Regenerative Strategies Targeting Angiogenesis in Flaps

### 3.1. Molecular Pro-Angiogenic Therapies

#### 3.1.1. Direct Pro-Angiogenic Factors

**Figure 2:**
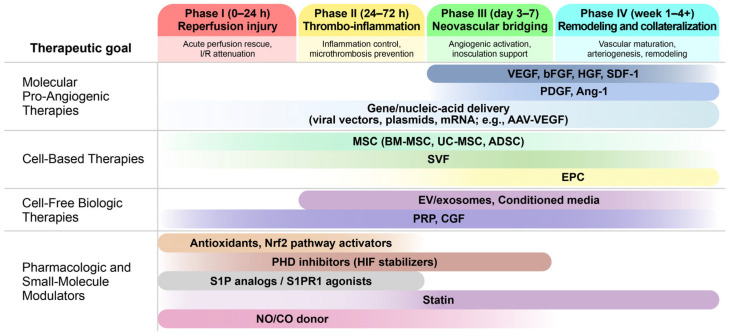
The dominant therapeutic intent and the most relevant post-flap elevation time window for each modality are shown, acknowledging substantial mechanistic overlap across phases. For each modality, the primary window of action is depicted with darker shading, while secondary windows are indicated with lighter shading.

Direct administration of pro-angiogenic factors, through recombinant proteins or topical formulations, aims to transiently enhance endothelial activation and neovascular sprouting during the critical ischemic window following flap elevation [76,77] (Figure 2). Despite a strong biological rationale, direct factor therapy is limited by short tissue residence time and diffusion-restricted distribution, which can create steep concentration gradients and result in insufficient exposure of distal regions [78,79].

VEGF is a key driver of early endothelial activation and sprouting through VEGFR-mediated signaling, promoting endothelial survival, migration, and tip-cell phenotypes. However, its effects on permeability can be a double-edged sword: transient leakiness may facilitate activation and remodeling, whereas excessive permeability exacerbates edema, increases interstitial pressure, and impairs microcirculatory flow, particularly in marginal distal regions [42,80]. bFGF (FGF2) functions as a pleiotropic enhancer of early angiogenesis by delivering potent mitogenic and pro-migratory signals to endothelial cells and facilitating matrix permissiveness through protease activation and stromal remodeling. Functionally, it supports the proliferative expansion and morphogenesis of nascent vascular networks, complementing VEGF-driven sprouting [42,77,81].

In contrast, PDGF and Ang-1 are more closely associated with vessel maturation and stabilization. PDGF signaling promotes the recruitment and maintenance of mural cells, supporting lumen integrity and durable perfusion, and may secondarily reinforce pro-angiogenic remodeling programs in ischemic tissue [76,82]. Ang-1, through Tie2 signaling, strengthens endothelial junctions and fosters a stabilized vascular phenotype, often regarded as a counterbalance to the excessive leakiness observed in newly formed microvessels [76].

HGF contributes a unique dimension via c-Met signaling by integrating angiogenic, cytoprotective, and motogenic effects. Importantly, pro-angiogenic ligands vary in their effects on barrier function and inflammation, which is clinically significant in flap procedures where the goal is to achieve functional reperfusion without exacerbating inflammatory injury [83,84].

SDF-1 (CXCL12) functions as a recruitment-based axis rather than following a classic growth factor paradigm. Through CXCR4-mediated chemotaxis, SDF-1 supports neovascularization by mobilizing and recruiting progenitor and reparative cells, thereby complementing growth factor-driven sprouting and remodeling [85,86].

#### 3.1.2. Gene/Nucleic Acid-Based Delivery of Angiogenic Factors

Gene and nucleic acid approaches in flap models are employed to modulate the onset, duration, and spatial distribution of pro-angiogenic signaling within ischemia-relevant compartments [79,87,88]. Therefore, platform selection should be evaluated based on expression kinetics, compartment-level coverage, and the feasibility of re-dosing, as these factors often influence efficacy as much as the choice of cargo [89].

Flap-directed delivery methods can be categorized into plasmid-based delivery, viral vectors, and cell-mediated gene delivery. In pooled VEGF datasets, plasmids, viruses, and cells have all been utilized; however, subgroup differentiation by vector type has not been observed. This pattern highlights that cross-study interpretation is often limited when core delivery descriptors—particularly perioperative timing, route, dose, and tissue-level distribution—are not reported in a standardized manner [90]. VEGF remains the most commonly used prototypical cargo in flap-directed gene delivery, while other targets, such as hypoxia-inducible factor (HIF), angiopoietin-1 (Ang-1), and SDF-1, have been reported less consistently [86,91,92]. Beyond VEGF-driven sprouting, cargo selection can be employed to target vascular stabilization or broader hypoxia-adaptation programs. Ang-1 is commonly characterized as a Tie2-mediated stabilization signal that may counteract hyperpermeability, whereas upstream regulators such as HIF-1α aim to activate coordinated survival pathways but require precise spatial and temporal control to minimize off-target angiogenesis [91,92,93,94]. Across models, local tissue injection remains the predominant delivery route, whereas intra-arterial, intrafascial, and topical routes are too sparsely represented to support robust route-specific inferences and are therefore typically treated as rare-route outliers in pooled analyses.

### 3.2. Cell-Based Therapies

Cell-based strategies aim to enhance peri-flap angiogenic remodeling by introducing living signal sources that respond to ischemia, inflammatory activation, and extracellular matrix cues. In flap contexts, their primary function is to shift the local angiogenic program toward coordinated reperfusion recovery by supporting endothelial cell viability and remodeling, while mitigating maladaptive inflammatory and stromal responses during the ischemic and early remodeling phases [95,96,97].

Bone marrow-derived mesenchymal stem cells (BM-MSCs) represent a canonical source with extensive preclinical experience; however, bone marrow harvesting is invasive and yields relatively low numbers of stromal cells per volume. These practical limitations have driven interest in adipose-derived stromal cells (ADSCs) and the stromal vascular fraction (SVF), which can be obtained in larger quantities and provide a heterogeneous, interactive cell population. This population includes stromal cells, endothelial-lineage cells—often described as endothelial progenitor cells (EPCs) or endothelial colony-forming cells (ECFCs)—and mural cell progenitors [97]. Umbilical cord-derived MSCs (UC-MSCs) have also been evaluated in flap-related preclinical studies and are frequently considered candidates for allogeneic, scalable manufacturing due to their high proliferative capacity and low donor-site morbidity [96].

A prevailing working model conceptualizes cell-based therapy as microenvironmental regulation, wherein efficacy is primarily mediated by secreted factors and vesicular cargo rather than by durable structural engraftment [95,97]. In SVF- and ADSC-centered paradigms, pro-angiogenic and pro-repair mediators include VEGF, HGF, FGF2, and angiopoietin-family signals, accompanied by immune- and matrix-regulatory cytokines that collectively modulate endothelial activation, matrix permissiveness, and tissue stress responses [97]. These paracrine programs likely concentrate their impact during the early post-ischemic window by influencing both the sprouting and subsequent maturation of fragile neo-vessels. Functionally, these programs are expected to be most critical during the sprout-to-stability transition, where pericyte recruitment and basement membrane assembly determine whether nascent microvessels mature into durable conduits [98,99,100]. SVF descriptions incorporate this maturation logic by positioning ADSCs as perivascular support cells that promote vessel stabilization in concert with endothelial-lineage cells [97].

### 3.3. Cell-Free Biologic Therapies

Biologic and cell-free products occupy an intermediate position between single-factor delivery and live cell transplantation by providing concentrated regenerative bioactivity in an acellular format [101]. Within the phase framework of flap injury and repair, these products are conceptually aligned with early-phase microcirculatory protection and inflammatory modulation, while also supplying regenerative cues that support the subsequent neovascular bridging window. Their practical appeal in flap surgery lies in their ability to be applied locally to ischemic tissue, potentially reducing the logistical and safety challenges associated with preparing viable cell products [3,101]. However, successful translation depends on standardized product definitions and potency characterization, as variability in preparation protocols can significantly affect reproducibility [102,103].

Secretome-derived preparations include extracellular vesicle (EV) or exosome-enriched fractions, as well as conditioned media, which represent distinct yet related paracrine product formats. EVs, including exosomes, microvesicles and apoptotic bodies, serve as intercellular messengers by encapsulating bioactive cargo such as proteins, lipids, and regulatory RNAs [104,105]. Because EV cargo reflects the cell of origin and the context of biogenesis, EV signaling is inherently context-dependent and can modulate endothelial activation, inflammatory restraint, and survival programs that are collectively required in flap ischemia [106,107,108]. Cross-study interpretation is limited by heterogeneous EV isolation and characterization methods, as well as inconsistent dosing units, such as particle number versus protein mass.

Platelet-derived products offer a clinically established, autologous method to enhance early repair signals. Platelet-rich plasma (PRP) and related formulations provide a concentrated source of platelet-derived mediators that regulate endothelial activation, stromal remodeling, and inflammatory balance. This aligns with the early flap phase, which is characterized by microcirculatory instability and oxidative stress. Successful clinical application depends on preparation protocols and quality control, as factors such as platelet concentration, leukocyte content, and activation method can significantly influence biological outcomes [103,109,110].

### 3.4. Pharmacologic and Small-Molecule Modulators

Pharmacologic and small-molecule modulators represent a diverse yet clinically practical class of adjunctive interventions that enhance flap viability by preserving functional perfusion and directing the ischemic tissue response toward repair. For clarity, these agents are categorized into three overlapping mechanistic themes: redox regulation, hypoxia-adaptive and endothelial-stabilizing signaling, and gasotransmitter-based modulation [111,112,113,114,115]. Compared to proteins or cellular products, small molecules are generally easier to dose and incorporate into perioperative workflows; however, successful translation requires balancing local efficacy with potential pleiotropic off-target effects on hemodynamics, coagulation, and systemic immunity [3,116].

One focus is on redox regulation and cytoprotection. By reducing the burden of ROS and preserving the viability of endothelial and parenchymal cells, antioxidant mechanisms can indirectly enhance microvascular stability and create a favorable environment for angiogenic remodeling [117]. Biliverdin serves as a representative example, proposed to mitigate oxidative stress and apoptosis while promoting angiogenic responses through PI3K–Akt-mediated activation of the Nrf2 antioxidant pathway [118]. In this context, the primary benefit of redox-targeted therapy is not to directly induce a “pro-angiogenic switch,” but rather to prevent early microvascular dysfunction caused by edema, barrier leakage, and cell loss, which otherwise limit effective reperfusion recovery [59,117].

A second approach targets hypoxia-adaptive signaling and endothelial stabilization. Prolyl hydroxylase domain (PHD) inhibitors can enhance hypoxia-responsive transcriptional programs, including VEGF and broader survival mediators, thereby increasing angiogenic competence and stress tolerance in compromised tissues [68,94,119,120]. Complementary strategies focus on maintaining endothelial barrier integrity and junctional organization. Since sphingosine-1-phosphate (S1P) receptor signaling regulates endothelial survival pathways and adherens junction dynamics, S1P analogs and S1PR1 agonists can be employed as interventions that shift the microvasculature toward a less permeable, more functionally perfused state, thereby complementing angiogenesis-oriented approaches [121,122]. Statins represent a clinically accessible class frequently repurposed in ischemic tissue paradigms, with proposed mechanisms including improved endothelial function, autophagy-associated cytoprotection, and attenuation of oxidative stress and apoptosis, along with secondary reinforcement of pro-angiogenic signaling depending on the context [123,124,125]. Clinically, statins are appealing as a low-burden perioperative adjunct for vasculopathic conditions such as diabetes, smoking, or radiation-induced tissue damage. However, their effectiveness is likely limited when flap compromise results from mechanical inflow or outflow failure [124].

A third axis involves gasotransmitter-based strategies centered on nitric oxide (NO) and carbon monoxide (CO). These endogenous mediators exert concentration-dependent effects on vascular tone, redox balance, platelet–leukocyte interactions, and endothelial signaling, thereby directly influencing microvascular patency and inflammatory containment [111,126,127,128]. Mechanistically, the heme oxygenase-1 (HO-1) pathway provides an integrative framework linking CO production with biliverdin and bilirubin generation, coupling vasoregulatory and antioxidant functions that are critical for ischemic tissue protection and reparative angiogenesis [111,129,130]. Concurrently, NO and CO suppress hypoxia-induced VEGF transcription by reducing HIF-1 binding at the VEGF 5′ hypoxic enhancer [128].

### 3.5. Biomaterial Platforms for Angiogenesis in Flaps

Biomaterial platforms function as enabling technologies that modulate the peri-flap microenvironment by controlling the location and duration of angiogenic stimulus presentation [131]. Compared to bolus administration of labile factors, engineered matrices can extend the local availability of cues, protect cargo from degradation, and impose spatial constraints that guide endothelial organization [132]. These capabilities are especially important in flap surgery because perfusion deficits are both territorial and time-dependent, and the distal microcirculation is highly sensitive to edema, inflammatory activation, and heterogeneous integration with the recipient bed [6].

#### 3.5.1. Injectable Depots and Patches: Hydrogels and Adhesive Gels

Injectable depots and patch-type hydrogels are designed to reduce rapid clearance and mitigate unfavorable diffusion gradients toward distal regions by enhancing local retention and prolonging exposure within the target compartment following simple injection or placement [133,134]. Tissue-adhesive formulations provide mechanical fixation on wet tissue surfaces, which is particularly relevant when shear forces, edema, or exudate might otherwise displace the material. Although much of the supporting evidence comes from wound models, the primary design considerations—including exposure timing, local barrier conditions, and hypoxia-associated remodeling—overlap with flap border-zone biology and are therefore relevant for flap-directed delivery.

Photocrosslinked gelatin hydrogels prepared using visible light irradiation have been reported to adhere rapidly to wet tissue surfaces, maintain biocompatibility, and provide sustained bFGF release. In these systems, prolonged local availability of bFGF coincided with improved wound healing and increased survival in skin flap models. The conformability of gelatin-based depots was also noted to facilitate extended contact with uneven tissue surfaces during factor delivery [133].

Self-healing injectable hydrogels based on dynamic coordinative crosslinking have been developed to enhance injectability and durability while minimizing swelling compared to conventional formulations. This property may reduce pressure-related microcirculatory compromise in confined tissue compartments. A multifunctional polyethylene glycol hydrogel incorporating mangiferin liposomes has been reported to provide sustained release and is associated with improved neovascularization and reduced hypoxia-induced apoptosis, with links to Bax/Bcl-2/caspase-3 regulation. In random skin flap models, the same formulation reduced necrosis and exhibited anti-inflammatory and anti-infective effects alongside pro-neovascularization activity [134,135].

Tissue-adhesive hydrogel systems incorporating growth factor-loaded secondary carriers combine strong adhesion with sustained release from liposomal depots. Hydrogels composed of human serum albumin and o-phthalaldehyde-functionalized four-arm polyethylene glycol have been shown to exhibit robust tissue adhesion, biodegradability, and biocompatibility, while enabling prolonged release of bFGF from embedded liposomes [136]. In full-thickness skin incision models, these hydrogels improved wound closure and healing outcomes compared to commercially available fibrin glue and cyanoacrylate adhesives, with histological findings consistent with increased angiogenic activity [137].

An endogenous-first strategy employs injectable matrices to modulate the immune and stromal microenvironments rather than delivering exogenous proteins. Macrophage-activating hydrogels composed of konjac glucomannan and heparin have been developed to enhance macrophage-derived production of pro-angiogenic signals while sequestering locally produced factors through heparin moieties. These hydrogels have been reported to increase vessel formation and maturation following implantation [138]. Related approaches include gelatin methacrylate hydrogels incorporating autologous concentrated growth factor (CGF), which exhibit plasticity, adhesive properties, injectability, and self-healing behavior while limiting rapid factor release and degradation. These materials have been associated with improvements in vascularization-related outcomes, supported by corresponding matrix remodeling and re-epithelialization results [139].

Depot systems have also been investigated for payloads that act upstream of angiogenesis by modulating vasomotor tone and oxidative stress. In flap models, thermosensitive hydrogels loaded with papaverine have been shown to improve flap survival area and reduce edema. These effects are supported by biochemical and vascularization indicators, including increased superoxide dismutase activity, decreased malondialdehyde levels, higher mean vessel density, and upregulation of CD34 and VEGF [140]. Similarly, hydrogel depots have been employed for nucleic acid delivery, including extracellular vesicle-associated VEGF mRNA, which has been reported to enhance flap viability and vascular regeneration. Transcriptomic profiling revealed upregulation, relative to controls, of angiogenesis-related gene sets, including the VEGF pathway, as well as pathways involved in collagen remodeling and anti-oxidative stress responses [11].

#### 3.5.2. Solid Scaffolds: Electrospun Nanofibers and Porous 3D Scaffolds

Solid scaffolds offer structural guidance and stable interfaces for cell attachment while serving as reliable reservoirs for bioactive cues [141,142,143]. These platforms are particularly useful when a prolonged presentation, spatial compartmentalization, or staged exposure of signals is required, beyond simple local retention [142,144,145]. Despite the evidence base being drawn primarily from excisional wound models, these scaffold characteristics are directly applicable to flap procedures that demand durable structural support and spatially confined cue presentation, including border-zone remodeling, inosculation, and prefabrication [146,147,148].

Electrospun nanofibers mimic the fibrillar architecture of the extracellular matrix (ECM) and provide a high surface area for the attachment of affinity ligands and the loading of growth factors [149,150]. Poly(ε-caprolactone)/gelatin nanofibers co-functionalized with the laminin-derived YIGSR peptide and heparin enhanced endothelial functions relevant to angiogenesis, with mechanistic links to the FAK–MAPK/ERK signaling pathway, and were associated with accelerated wound closure in murine dorsal skin defects [151]. Composite nanofiber constructs have also been engineered for staged, multi-factor release (VEGF, PDGF, bFGF, EGF) using collagen–hyaluronic acid matrices incorporating gelatin nanoparticles, enabling early epithelial coverage and sprouting, followed by later maturation-phase responses [152]. Beyond passive release, spatiotemporally patterned delivery has been achieved using near-infrared irradiation through photomasks to trigger phase-specific release of PDGF-BB, VEGF, and EGF, resulting in enhanced vascularization and remodeling in porcine wound models [153].

Porous 3D scaffolds feature open macropore networks that facilitate cell infiltration and support the transport of oxygen and nutrients, which are essential for stable neovascularization in thicker constructs. Key architectural parameters, including pore size, interconnectivity, spatial distribution, and micro/nano-topography, have been shown to influence endothelial behavior and vessel formation [154]. In flap-oriented construct engineering, collagen–chitosan scaffolds seeded with ADSCs and impregnated with VEGF-containing poly(lactic-co-glycolic acid) (PLGA)/poly(ethylene glycol) (PEG) microspheres were implanted around a vascular pedicle, promoting neovascularization and enhancing soft-tissue persistence [131]. More advanced constructs, such as 3D-bioprinted engineered tissue flaps with hierarchical vessel networks (VesselNet), have been developed for direct host-to-implant perfusion via surgical anastomosis, with functional integration confirmed by contrast microcomputed tomography and lectin perfusion [155]. Macroporous silk fibroin scaffolds with nanofibrous microstructures have also been linked to improved neovascularization and dermal reconstruction, as evidenced by infiltration and matrix remodeling in wound models [156].

#### 3.5.3. Particulate Carriers: PLGA Microspheres and Nanoparticles, Lipid Nanoparticles, Inorganic Nanocapsules

Particulate carriers are used to protect labile pro-angiogenic cargo, prolong local exposure, and modulate release kinetics, including staged or near zero-order profiles [157,158]. These properties are particularly relevant to flap biology because both insufficient exposure and excessive, poorly localized exposure can be detrimental, especially when increased permeability and edema contribute to microcirculatory compromise [9,159].

PLGA microspheres and nanoparticles provide sustained, controlled release while minimizing the degradation of encapsulated factors. Coacervate-coated PLGA systems co-delivering VEGF and TGF-β3 have been reported to reduce necrosis and improve perfusion in mouse skin flap models, consistent with additive or synergistic effects from dual-cue exposure [160]. IGF-loaded PLGA microspheres have been shown to promote angiogenesis and accelerate skin flap repair, potentially through reduced oxidative stress and activation of the Ang1/Tie2 pathway [161]. Release kinetics have also been engineered at the material level; multiscale microspheres with nanostructured silicon cores and PLGA outer shells were designed for near zero-order PDGF-BB release and were associated with localized neovascularization and expanded functional vessel coverage over time [157]. Hybrid constructs have integrated particles into patches—for example, electrospun patches functionalized with PLGA–porous silica nanoparticles to enable spatiotemporal VEGF and PDGF-BB release—resulting in increased vascular markers such as α-SMA and CD31, as well as enhanced expression of angiogenesis-associated genes [162]. Multi-cargo strategies have also been described, including active self-encapsulation that enables simultaneous loading and multi-week release of VEGF, FGF, and IGF, supporting sustained co-exposure without repeated dosing [163].

Lipid nanoparticles (LNPs) facilitate intracellular delivery of mRNA-based angiogenic therapies, enabling transient expression without genomic integration. Ionizable LNPs encapsulating VEGFA mRNA have demonstrated high transfection efficiency and effective endosomal escape. Treated wounds exhibited accelerated closure and increased vascular density, accompanied by enhanced epithelialization and matrix remodeling [164]. In diabetic wound models, LNP-delivered VEGF-A mRNA improved closure kinetics and increased endothelial proliferation compared to controls [165]. Modified VEGF-A mRNA delivered via LNPs induced dose-dependent vasodilation, increased blood flow, and neovessel formation, with sequential dosing further enhancing tissue oxygenation during diabetic wound healing [166]. Additionally, LNP-based strategies have been proposed to extend mRNA distribution through extracellular vesicles (EVs) as intermediates; cardiac progenitor cell-derived EVs have been reported to promote angiogenesis while minimizing inflammatory cytokine induction [167].

Inorganic nanoparticles, including nanoporous silica and gold nanoparticles, introduce design parameters related to surface chemistry, affinity loading, and ion-mediated signaling [168]. Nanoporous silica nanoparticles loaded with VEGF via amino-group interactions have been reported to enable long-term release for up to 100 days, with the released VEGF inducing tube formation comparable to repeated dosing with soluble VEGF [169]. In flap-directed applications, prolonged release can reduce the need for re-dosing; however, long-term persistence, clearance, and tissue compatibility become primary translational challenges [170]. Mesoporous silica microcarriers releasing both silicon ions and VEGF have been shown to enhance endothelial migration and network formation, with silicon-ion–linked HIF-1α upregulation proposed as a contributing mechanism [171]. Nanosilicates have been reported to promote angiogenesis through ROS-mediated activation of the WNT/β-catenin pathway [172]. Gold nanoparticles exhibit context-dependent effects; peptide-capped formulations have been shown to induce endothelial responses via receptor-mediated interactions [173,174].

#### 3.5.4. EV Delivery and Spatially Patterned Bioactive Coatings

In flap applications, EV-based interventions are constrained by rapid dispersion and clearance within territorially ischemic compartments, making local residence time and positional confinement critical determinants of bioavailability [175]. Recent work further indicates that delivery format can be as important as EV identity in determining effective exposure in vivo. Platelet-lysate small EVs improved survival and angiogenesis markers, and a thermosensitive sprayable gel formulation increased retention with a detectable local signal maintained through POD7, aligning residence time with the early remodeling window [176]. Consequently, delivery matrices are employed to enhance intratissue retention and sustain effective exposure in distal regions, while spatially patterned coatings restrict cue presentation to specific interfaces, thereby promoting directional vascular ingrowth and minimizing diffuse exposure [175]. Representative implementations include thermosensitive hydrogel systems designed to prolong local EV retention during the early postoperative period, as well as antibacterial hydrogel platforms that localize EV-mediated nucleic acid delivery and pro-angiogenic signaling within prefabrication contexts [11,176].

Unlike depot-based delivery, which primarily increases overall stimulus levels, spatial patterning encodes positional information by restricting where cues are presented. This approach promotes directional sprouting and maturation from the recipient bed and potentially mitigates diffuse, permeability-associated edema. Spatially controlled coating of Caf1-YIGSR and Caf1-VEGF on 3D porous hydrogels was employed to separate adhesion and proliferation signals (YIGSR) from migratory and sprouting cues (VEGF) [177,178]. Photopatterning of VEGF within collagen–glycosaminoglycan scaffolds using benzophenone photolithography induced spatially confined endothelial responses, with immobilized VEGF associated with increased infiltration and immature vascular network formation [179]. Acoustically responsive scaffolds enabled ultrasound-controlled bFGF release, where spatially defined acoustic droplet vaporization elicited corresponding in vivo angiogenesis patterns [180]. Elastin-like recombinamer scaffolds combining tunable proteolytic sequences with VEGF-mimetic peptides provided spatiotemporal control, with proteolytic rate and peptide activity jointly shaping infiltration and capillary formation [181]. MMP-sensitive PEG diacrylate hydrogels incorporating gradients of modulus, degradation, and YRGDS ligands were reported to stimulate directional vascular sprouting, with longer sprout extension aligned parallel to the gradient [182].

## 4. Preclinical and Clinical Evidence for Angiogenesis-Targeted Flap Support

Clinical evidence supporting angiogenesis-oriented adjuncts in compromised flaps remains limited and primarily stems from small, often uncontrolled series that report gross viability outcomes [183,184]. Consequently, the mechanistic and quantitative foundation of this field is largely based on experimental in vivo flap models conducted under controlled ischemic conditions. For this narrative synthesis, evidence was primarily identified through a targeted PubMed search of studies published up to January 2026, supplemented by citation screening of relevant reviews and seminal articles. We included flap-specific preclinical in vivo models and clinical flap surgery studies evaluating angiogenesis-oriented adjuncts that reported survival or necrosis outcomes and, when available, objective perfusion or microvascular endpoints. To ensure the evidence remained focused on flap-specific territorial ischemia and to facilitate cross-study comparisons, we prioritized established flap models with clearly defined ischemic territories and well-documented dosing and timing. Non-flap wound models were excluded. In the following sections, experimental evidence is synthesized by therapeutic class, with available clinical observations briefly summarized within the relevant cell-free biologics subsection.

Comparative mechanistic analysis reveals that protein therapy offers immediate bioactivity but requires controlled-release formulations for durability; gene therapy provides sustained, high-level expression of single or combined growth factors with dose-dependent angiogenic effects; cell-based therapy delivers multimodal paracrine, immunomodulatory, and regenerative effects; exosome-based therapy delivers cell-free, multifactorial paracrine cargo that couples pro-angiogenic signaling with immunomodulation, reducing the logistical and safety burdens of live-cell transplantation; platelet-derived products provide balanced, autologous multi-factor delivery with anti-inflammatory properties; and pharmacological agents provide immediate bioactivity and multi-target effects but often require repeated dosing or controlled-release formulations for sustained benefit, while gasotransmitter strategies offer rapid vasodilatory and angiogenic effects, with biomaterial-based delivery systems enabling tunable and localized release [3,37,90,96,185,186,187]. The choice of modality depends on clinical context, with combination strategies increasingly explored for synergistic effects on flap survival and vascularization.

Many preclinical studies report increased capillary density or elevated endothelial markers; however, these indicators do not necessarily reflect functional reperfusion. Accordingly, this review discusses angiogenesis-related measures alongside endpoints that assess blood flow, tissue oxygenation, barrier dysfunction or edema, and venous drainage when such data are available.

### 4.1. Molecular Pro-Angiogenic Therapies

#### 4.1.1. Direct Pro-Angiogenic Factors

Protein delivery provides immediate bioactivity, enabling perioperative modulation of flap perfusion without relying on durable engraftment. Across studies involving VEGF protein, outcomes vary depending on delivery timing, route, and tissue compartment. For example, intra-arterial recombinant VEGF administered during surgery improved postoperative day 7 (POD7) survival and vascular parameters compared to saline [80] (Table 1). In a dorsal random-pattern model, pre-elevation subcutaneous delivery enhanced distal survival more effectively than delivery to the recipient-bed fascia, while intraoperative fascial delivery exhibited the weakest effect [188]. These findings indicate that the timing and location of factor presentation can be as influential as the ligand’s identity.

Beyond VEGF, other proteins also exhibit schedule- and formulation-dependent effects. A four-day FGF preconditioning regimen reduced necrosis and increased vessel counts during early postoperative follow-up, with FGF-2 demonstrating the stronger effect [189]. Topical delivery of endothelial cell growth factor via a gelfoam carrier improved early viability and angiographic vascularization signals [190]. Additionally, SDF-1α delivered using gelatin microcarriers enhanced survival at POD7, with further benefits observed when combined with Matrigel coating [191]. PDGF-BB pretreatment was associated with near-complete survival and improved microvascular indices across flap segments [192].

#### 4.1.2. Gene/Nucleic Acid-Based Delivery of Angiogenic Factors

Gene and nucleic acid approaches aim to extend the exposure window beyond what is achievable with protein boluses; however, their efficacy depends on establishing timely transgene expression and effectively distributing the signal to ischemia-relevant compartments. A preclinical meta-analysis reported that VEGF gene delivery improved flap survival area by 15.66% compared to controls, whereas other gene-directed interventions showed smaller effect sizes, including PDGF (13.44%), VEGF + FGF (8.64%), HGF (5.61%), and FGF (3.84%) [90]. Across VEGF gene-delivery platforms, efficacy is highly context-dependent, varying according to flap type, delivery timing, and spatial targeting [87,88,193] (Table 2). Studies using AAV-VEGF165 demonstrate a timing-by-model interaction: dosing at flap elevation was most effective in an epigastric cutaneous flap, whereas preoperative intramuscular dosing yielded better results in a TRAM musculocutaneous flap [87]. Intradermal AAV regimens and adenoviral VEGF approaches generally reported improved viability-related outcomes compared to vector or saline controls, with some adenoviral designs showing enhanced effects following repeated preoperative [88,193]. Consistent with the importance of exposure geometry, injections targeted to ischemia-relevant regions more effectively reduced combined necrotic and hypoxic areas than less targeted delivery [78]. Additionally, non-viral plasmid strategies exhibited model-dependent sensitivity to early versus delayed administration when combined with electroporation [89]. Beyond VEGF, other transgenes have been evaluated and demonstrate target-specific outcome profiles. HIF-1α plasmid delivery reduced necrosis and increased CD31-based vessel density [92]. In direct comparisons, Ang-1 showed comparatively stronger effects on permeability-related measures than VEGF-focused regimens [91]. HGF gene therapy demonstrated beneficial effects using both viral and plasmid platforms, including improved survival and enhanced vascular indices based on perfusion and CD31 [84,194].

### 4.2. Cell-Based Therapies

In vivo flap models generally demonstrate a favorable efficacy signal for cell-based therapies, with reductions in necrosis and improvements in vascular outcomes. However, heterogeneity in flap design, dosing, and endpoints limits the ability to draw definitive conclusions across studies. A preclinical meta-analysis reported that stem cell treatment significantly reduced necrosis and increased both vessel density and VEGF expression compared to controls (necrosis: standardized mean difference [SMD] 3.20; vessel density: SMD 2.96; VEGF expression: SMD 4.34) [96].

Across BM-MSC studies, efficacy appears to be sensitive to both delivery timing and tissue compartment. Reported benefits include subcutaneous delivery in dorsal random-pattern flaps, dose-responsive effects following choke-zone injection in perforator designs, and post-reperfusion intravenous administration in ischemia–reperfusion models [195,196,197] (Table 3). Collectively, these findings suggest that the timing and location of cell delivery significantly influence cell viability and vascular outcomes. Additionally, in selected ischemia–reperfusion perforator models, engineered UC-MSCs further enhanced therapeutic effects; for example, F-5–transfected human umbilical cord-derived MSCs (hUC-MSCs) outperformed unmodified cells and controls [198].

Adipose-derived approaches demonstrate similarly favorable outcomes while emphasizing the effects of dose and formulation. Intraoperative intradermal delivery of autologous ADSCs improved viability in dorsal random-pattern flap models [199]. In contrast, an axial flap study involving arterial delivery revealed a non-linear dose–response relationship, with an intermediate dose providing the greatest benefit [200]. Comparative studies also suggest that SVF can outperform isolated ADSCs in certain contexts, and that matrix-supported delivery is more effective than suspension delivery at the recipient site, as evidenced by increased CD31-associated markers and pro-angiogenic signals [201,202]. Additionally, compartment-targeted SVF injection has been associated with measurable perfusion improvements in fascial flap models [203]. Beyond stromal cell preparations, EPC-based strategies have been evaluated in venous compromise models, where allogeneic EPCs enhanced survival alongside improvements in perfusion-related outcomes and endothelial signaling measures [204].

### 4.3. Cell-Free Biologic Therapies

Cell-free biologic adjuncts, particularly EVs, are consistently associated with improved viability in flap models, often accompanied by perfusion-related vascular readouts under ischemic stress. Across EV studies, efficacy commonly correlates with producer-cell conditioning and cargo composition. Conditioning paradigms such as oxidative or hypoxic preconditioning have been reported to enhance survival and perfusion signals compared to non-conditioned preparations [107,205] (Table 4). Mechanistic studies suggest that EV cargo can activate pathways involving HIF-associated vascular signaling, stress response and autophagy regulation, and modulation of inflammatory injury, with similar findings reported across EV sources, including platelet-derived preparations [108,176,206]. Comparative fractionation further indicates that vesicular and soluble paracrine fractions may provide overlapping benefits in some contexts, and IL-6 perturbation attenuated these effects [207].

Platelet-derived concentrates, particularly PRP and related preparations, consistently demonstrate positive effects across various experimental flap models [208,209,210,211]. Importantly, outcomes are highly dependent on the formulation and activation of PRP, as the procoagulant strategy and choice of carrier can influence results. For example, fibrin-based formulations reduce necrosis in a delay model, whereas thrombin alone exacerbates necrosis in the same context [212]. Additionally, the timing of administration relative to ischemia is critical; delivering PRP before or during ischemia yields greater improvements in tissue survival and perfusion than dosing after ischemia in ischemia–reperfusion models [213].

**Table 4:** Cell-Free Biologic Therapies in Preclinical In Vivo Flap Models: Representative Studies.

Study	Cell-Free Product	Model	Delivery Route, Timing, Dose	Control	Follow Up	Key Outcomes
Bai et al., 2018 [107]	ADSC-Exo vs. H_2_O_2_-ADSC-Exo	Rat; SIEA island flap (6 × 9 cm), I/R 6 h	SC; post-ischemia immediately; 100 µg in 200 µL, 6 sites	Veh (PBS, I/R)	POD5	Survival area (%) ↑ and perfusion ↑ * (H_2_O_2_-ADSC-Exo > ADSC-Exo *); CD31/MVD ↑ *; histologic injury ↓ *; apoptosis ↓ *.
Wu et al., 2022 [205]	ADSC-EVs (human ADSC-derived) vs. HT-ADSC-EVs (1% O_2_-conditioned)	Rat; modified McFarlane dorsal random skin flap (9 × 3 cm)	ID; IO; 10 µg in 0.2 mL, 6 sites	Veh (PBS)	POD7	Necrotic rate (%) ↓ (PBS 78.6 → ADSC-EVs 59.2 * → HT-ADSC-EVs 29.1 *); perfusion (PU) ↑ (354.7 → 556.3 * → 803.2 *); CD31+ cells and HIF-1α/VEGF↑ * (HT-ADSC-EVs > ADSC-EVs *).
Luo et al., 2024 [108]	M2-exo; mechanistic arm with 2-ME2	Mouse; modified dorsal random-pattern skin flap (5.5 × 1.5 cm)	Exo: IV; IO; 500 μg. 2-ME2: SC at flap edge; D0-POD3; 40 mg/kg/day	IV saline; M0-exo; vehicle control for 2-ME2 arm	POD7	Survival area ↑ * (86.2% vs. 47.7% control; IR estimate 85.5% vs. 52.2%); choke-zone angiogenesis ↑ (CD31+ vessels * 19.5 vs. 4.2; H&E microvessels * 20.3 vs. 4.3); benefit attenuated with HIF-1α inhibition (2-ME2).
Deng et al., 2023 [206]	Hypo-Exo; mechanistic arm with 3-MA	Rat; free inguinal skin flap (3 × 6 cm) with I/R (6 h ischemia)	Exo: ID; D0-POD7; 50 μg × 4 sites 3-MA: IP; D0-POD7; 10 mg/kg/day	I/R; Exo; sham	POD7	Survival area ↑ (Hypo-Exo > Exo * >I/R **); CD31+ vascularity ↑; ROS/inflammation/apoptosis ↓; 3-MA co-treatment abolished these benefits.
Pu et al., 2017 [207]	hADSCs vs. hADSC-CM vs. hADSC-Exo (IL-6 focus)	Mouse; long thoracic vessel-based pectoral skin flap (4 × 1 cm) with I/R (3 h ischemia)	Local inj. into flap	I/R + saline (vehicle); sham (no I/R)	POD5	Survival area ↑ * (ADSCs & ADSC-Exo vs. I/R control); microvessels ↑ * (ADSC 16.3 ± 1.9 vs. I/R 5.8 ± 1.4); IL-6 implicated (blocking/KO reduced benefit)
Liu et al., 2025 [176]	PL-sEV	Mouse; random-pattern skin flap	SC; IO; 100 µg/mL	Sham; Flap (untreated) ± PL	POD1–7	Necrosis ↓ * and temperature/thermal recovery ↑ * (PL-sEV > PL *); perfusion (LDPI) ↑ * with angiogenesis markers ↑ * (VEGF, CD34/CD31) and PANoptosis markers ↓ *.
	PLEL@PL-sEV (spray)	Mouse; random-pattern skin flap	Topical spray (in situ gelation); IO; 100 µg/mL	Flap; PLEL blank; PL-sEV	POD7	Retention ↑ (DiR signal detectable to POD7) vs. PL-sEV; survival/thermal preservation ↑ * vs. Flap/PLEL; epidermal/dermal structure preserved.
Sönmez et al., 2013 [208]	Strain-matched donor-derived PRP gel	Mouse; lateral thoracic artery pedicled axial skin flap (1.5 × 2.5 cm), I/R 4 h	Topical (undersurface); IO; 120 µL	Sham-OP; Isch (I/R)	POD14	Survival area (%) ↑ (PRP+Isch * 97.8 vs. PRP 87.8 vs. Isch 67.8 vs. Sham-OP 71.4); PRP higher vs. Isch/Sham-OP (NS).
Li et al., 2012 [210]	Autologous PRP; PPP	Rat; dorsal random skin flap (11 × 3 cm)	SC; IO; 25 µL × 4 sites	NoTx	POD7	Survival area (%) ↑ (PRP * 61.2 vs. PPP 35.8 vs. NoTx 28.0); vessel density and VEGF/PDGF transcripts ↑ *.
Chai et al., 2019 [211]	PRP gel	Rat; bilateral dorsal skin flap (2.5 cm diameter; 1 × 1 cm pedicle)	Topical (undersurface); IO; 1 mL/10 cm^2^	NoTx	POD1, 3, 5, 7	Survival area (%) ↑ POD5 * (70.5 vs. 61.1) and POD7 * (51.3 vs. 33.0) (PRP vs. NoTx); improved remodeling.
Rah et al., 2017 [209]	Strain-matched donor-derived PRP	Mouse; lateral thoracic artery axial island flap (1.5 × 3.5 cm), I/R 4 h	SC (under flap); IO; 120 µL	PBS; PBS + I/R	POD1,3,5,7,10	Survival area (%) ↑ POD3–10 * (numeric NR). Perfusion (BPU ratio) ↑ at POD1 * and POD5 *; I/R+PRP > I/R at POD1 * and POD5 *.
Findikcioglu et al., 2012 [212]	PRP (irradiated, non-autologous); PPP+thrombin (FG); Thrombin	Rat; bilateral SIEA abdominal island flap; surgical delay (pedicles ligated day 5)	Topical (under flap); IO; (before closure); PRP or PPP 1.25 mL, thrombin 0.125 mL	Contralateral flap (NoTx)	POD7 (from pedicle ligation)	Necrotic area (%) ↓ PRP * (14.8 vs. 22.5 contralateral control) and FG * (18.1); thrombin-only ↑ necrosis (61.8) *; ↑ small-vessel neovascularization (PRP > FG > thrombin).
Su et al., 2024 [213]	Strain-matched donor-derived PRP	Mouse; lateral thoracic artery axial skin flap (2.5 × 4 cm), I/R (4 h Isch, 12 h reperfusion)	SC; Day 0 (pre (0 h), mid (4 h), post (10 h); 150 µL	PBS; PBS + I/R	POD1,4, 7	Survival area (%) ↑ at POD4,7 and Perfusion at POD1–7 ↑ with pre- & mid-PRP * vs. PBS; HIF-1α/VEGF ↑ * (pre-PRP strongest); Oxidative stress & anti-oxidant enzymes ↑ in I/R

* *p* < 0.05; ** *p* < 0.01; ↑ increase; ↓ decrease; ADSC, adipose-derived stromal/stem cell; BPU, blood perfusion unit; CD31, cluster of differentiation 31; CD34, cluster of differentiation 34; DiR, near-infrared lipophilic fluorescent tracer; EV, extracellular vesicle(s); Exo, exosome(s); FG, fibrin gel; hADSC, human ADSC; Hypo-Exo, Hypoxia-conditioned MSC exosomes; HIF-1α, hypoxia-inducible factor 1 alpha; HT, hypoxia-treated; H_2_O_2_, hydrogen peroxide; ID, intradermal; IO, intraoperative; I/R, ischemia–reperfusion; Isch, ischemia (group label); LDPI, laser Doppler perfusion imaging; M2-exo, M2 macrophage-derived exosomes; MVD, microvessel density; NO, nitric oxide; NoTx, no treatment; NR, not reported; O_2_, oxygen; OP, operation; PBS, phosphate-buffered saline; PDGF, platelet-derived growth factor; PL, platelet lysate; PL-sEV, platelet lysate–derived small extracellular vesicle(s); PLEL, PDLLA-PEG-PDLLA thermosensitive hydrogel; POD, postoperative day; PPP, platelet-poor plasma; PRP, platelet-rich plasma; PU, perfusion unit; SC, subcutaneous; sEV, small extracellular vesicle(s); SIEA, superficial inferior epigastric artery; SOD, superoxide dismutase; TEM, transmission electron microscopy; Veh, vehicle; vs., versus; 2-ME2, 2-methoxyestradiol.

### 4.4. Pharmacologic and Small-Molecule Modulators

Biliverdin exemplifies redox-directed cytoprotection, demonstrating enhanced survival and perfusion alongside concurrent changes in vascular-associated parameters and tissue injury indices [118] (Table 5). Modulation of the hypoxia pathway through PHD inhibition consistently demonstrates efficacy. Dimethyloxalylglycine (DMOG) reduced distal necrosis and improved perfusion across multiple studies involving perioperative and early postoperative schedules, with dose-dependent effects observed in some designs [68,94,119]. Similarly, Roxadustat (FG-4592) preconditioning enhanced survival and choke-zone perfusion, accompanied by increases in HIF-1α and inducible nitric oxide synthase (iNOS)-associated markers, as well as tissue NO indices [214].

Barrier-oriented modulation has been investigated through S1P receptor signaling. SEW2871 treatment increased survival compared to untreated and vehicle controls, accompanied by higher CD31-associated microvessel density, elevated levels of VEGFA and bFGF, and reduced tissue injury signals [215].

Statins have been extensively evaluated in flap models. Pravastatin and rosuvastatin improved survival and perfusion, accompanied by reductions in edema and markers of inflammatory injury; one study suggested a partial dependence on AMPK-linked autophagy signaling [123,125]. In a diabetic context, atorvastatin enhanced survival and capillary density and was associated with increased circulating EPCs and EPC recruitment to the flap [124].

Finally, gasotransmitter-related strategies have been evaluated using precursor supplementation and localized delivery systems. Dietary nitrate improved survival and distal perfusion, increased microvessel density, and reduced inflammatory cytokines [127]. A dual-gas hydrogel patch delivering CO and NO improved early perfusion and survival in an ischemia–reperfusion model and also increased arteriovenous fistula (AVF) patency by reducing intimal hyperplasia [126].

### 4.5. Clinical Evidence and Translational Safety Considerations

Clinical evidence in flap surgery remains limited and primarily derives from small, uncontrolled case series. In skin flaps affected by degloving injuries, local injection of autologous PRP was associated with an average survival rate of approximately 75%; however, this study lacked a control group [184]. Another small series investigating flap ischemia–reperfusion injury reported a 94.5% survival rate at day 7 following autologous CGF injection administered after clinical recognition of injury [183]. Due to the single-arm design and heterogeneous clinical contexts, these findings should be considered hypothesis-generating rather than conclusive evidence of efficacy.

Beyond the scarcity of clinical data, the clinical application of vascular regenerative strategies remains limited by several practical and safety concerns. In post-resection reconstruction, oncologic safety is a critical consideration because the same pro-angiogenic pathways that promote neovascularization may theoretically also support residual tumor cells [216]. Although flap-specific clinical datasets are limited, follow-up data from VEGF-based therapies in cardiovascular indications have not demonstrated a clear malignancy signal to date in small cohorts [217,218]. Nevertheless, candidates should be screened for malignancy or premalignant conditions, and ongoing oncologic surveillance should be considered. Another concern is the risk of excessive or aberrant angiogenesis. Uncontrolled VEGF expression can lead to disorganized, leaky vasculature with inadequate pericyte coverage, potentially resulting in hemangioma-like structures rather than functional capillary networks. This risk appears to depend more on local VEGF concentration and spatial distribution than on the total dose, effectively defining a tissue-specific therapeutic window [219,220]. Accordingly, delivery strategies should be designed to maintain exposure within this window and, when necessary, be complemented by signals that promote vessel stabilization [219].

The timing of administration is also critical across different modalities. In gene-delivery platforms, outcomes vary depending on flap type, delivery route, and whether dosing occurs before flap elevation or during surgery [89,90]. In cell therapy studies, preconditioning regimens sometimes yield better results than intraoperative delivery, whereas in ischemia–reperfusion models, interventions during reperfusion can be more effective than earlier dosing in certain contexts [96,221,222]. For vector-based approaches, additional safety concerns include the risk of insertional mutagenesis with integrating vectors, dose-dependent hepatotoxicity, immunogenicity against viral capsids, and the potential for complement-mediated thrombotic microangiopathy [223,224,225]. Although ex vivo transgenic cell strategies reduce delivery-related risks by limiting direct host exposure to viral components, clinical implementation still depends on maintaining transgene expression for several weeks to support vessel stabilization and achieving sufficiently homogeneous cell distribution throughout the three-dimensional flap tissue [219,226].

Addressing these challenges will require a combination of strategies that balance pro-angiogenic stimulation with vessel maturation signals, such as pairing VEGF with PDGF-B; the implementation of controlled-release delivery systems to maintain therapeutic concentrations within a safe window; the development of non-integrating or self-inactivating vectors to reduce genotoxicity; and rigorous prospective trials with standardized endpoints [219,227,228,229,230]. These approaches are essential to translate preclinical promise into safe and effective clinical applications for compromised flaps [227,228].

## 5. Summary and Key Insights

Angiogenesis-oriented adjuncts improve flap outcomes only when they restore hemodynamically competent microcirculation in territorially ischemic tissue. Across modalities, efficacy consistently depends on (i) spatiotemporal exposure control aligned with the ischemic and early remodeling windows, (ii) compartment-level coverage that reaches distal and border zones without provoking diffuse edema, and (iii) maturation and outflow competence—including mural cell support, barrier integrity, and venous drainage—that convert neovessels into functional perfusion.

Direct pro-angiogenic factors are suitable for perioperative use due to their immediate bioactivity; however, their short residence time and diffusion-limited spread make controlled release and compartment targeting essential to avoid permeability-driven edema. Gene and nucleic acid delivery extend the exposure window by modulating onset, duration, and spatial distribution, yet outcomes are highly context-dependent. Translation requires safety-conscious design to mitigate off-target angiogenesis, immune activation, persistence, and re-dosing constraints. Cell-based therapies act as multimodal regulators by coupling angiogenic cues with immunomodulation and matrix remodeling; successful translation depends on standardized manufacturing and potency metrics, as well as linking therapeutic benefit to functional perfusion and maturation endpoints rather than capillary surrogates alone. Cell-free biologics—such as EVs and platelet-derived products—offer multifactorial paracrine benefits with fewer logistical challenges than live cells, but efficacy varies with donor state and processing variables, making clear product definitions, dosing units, and potency assays essential. Pharmacologic modulators and gasotransmitter strategies are workflow-friendly and rapid; however, their durability and pleiotropic effects necessitate objective assessments of perfusion, edema, or barrier function, along with systemic safety considerations in vasculopathic or post-resection settings. Biomaterial platforms provide biomimetic control over the spatial and temporal presentation of cues, improving retention and programmable release, and, through spatial patterning, adding positional information for directional ingrowth and staged maturation.

## 6. Conclusions

Acute flap compromise most often results from pedicle-level inflow or outflow issues and mechanical factors that require prompt recognition and re-exploration. However, even after restoring proximal patency, distal and border-zone survival may remain limited due to microcirculatory instability, thrombo-inflammatory obstruction, and incomplete vascular remodeling. Therefore, regenerative adjuncts targeting angiogenesis should be employed as complements to meticulous surgical technique and timely salvage procedures, enhancing resilience to ischemia–reperfusion injury while promoting stable, outflow-competent microvascular reperfusion.

Key translational barriers include biocompatibility in inflamed or irradiated tissues, swelling or pressure effects that reduce conductance, manufacturability at scale, and quantifiable tissue distribution to clarify the spatiotemporal exposure profile. Clinical deployment must also address risks such as off-target angiogenesis, immune activation, excessive persistence, and limited re-dosing options, particularly in vasculopathic and post-resection contexts. Future research should focus on three key areas. First, VEGF-centered gene therapies that incorporate spatiotemporal control and built-in safety mechanisms. Second, scalable paracrine products, such as MSC secretomes and extracellular vesicles, are supported by standardized dosing units and potency assays. Third, biomaterial platforms are designed to localize and sustain bioactivity while minimizing edema and maintaining barrier integrity and venous drainage. Progress will depend on standardized reporting of timing, administration route, dose, dosing units, and tissue distribution to ensure consistent interpretation of exposure across studies. Additionally, validation will require functional assessments of perfusion and microvascular stability under common clinical modifiers, including irradiation, infection, diabetes, and smoking.

## Figures and Tables

**Table 1:** Direct Pro-Angiogenic Factors in Preclinical In Vivo Flap Models: Representative Studies.

Study	Growth Factor	Model	Number of Flaps	Delivery Route, Timing, Dose	Control	Key Outcomes
Padubidri et al., 1996 [80]	rhVEGF	Rat, 10 × 10 cm epigastric skin flap	16 (8/group)	IA (epigastric artery); IO; 5 µg	Saline	-Survival area (%) at POD7 **: 71.9 vs. 53.7. -Laser Doppler blood flow: higher in the VEGF group, mainly in prox/mid zones at POD7. -Capillary density: higher in the VEGF group on histology.
Vourtsis et al., 2012 [188]	rVEGF164	Rat; dorsal random skin flap (1.5 × 7.5 cm)	45 (9/group)	Local inj.; D−7 SC (flap) or IO Fascia (RB) or IO SC (distal 1/3) or IO IntraFascia (flap); 10 µg/1 mL	Saline	-Survival area (%) at POD7 **: distal 1/3 SC 80.4 vs. saline 35.4; recipient-bed fascia 56.3; D−7 whole-flap SC 33.7; intrafascial 28.3. -CD34+ vessel density **: higher in recipient-bed fascia (168.33 vessels/mm^2^) and distal 1/3 SC (139.53 vessels/mm^2^) vs. saline (31.42 vessels/mm^2^). -Histology: increased angiogenesis, particularly in the recipient-bed fascia and distal 1/3 SC groups.
Fayazzadeh et al., 2016 [189]	FGF-1 (aFGF), FGF-2 (bFGF)	Rat; dorsal random-pattern skin flap (2 × 8 cm)	30 (10/group)	Preconditioning SC; D−4 to D−1 (4 daily inj.); 2.5 µg/day	Saline	-Necrosis (%) at POD10 **: control 41.1 vs. FGF-1 24.2 vs. FGF-2 7.7; FGF-2 vs. FGF-1 **. -Ischemic but viable area (%) at POD10: ** control 4.54 vs. FGF-1 25.6 vs. FGF-2 32.4; FGF-2 vs. FGF-1 *. -Total discolored area (%) at POD10 (necrosis + ischemia) (NS): 45.6 vs. 49.7 vs. 40.0. -Blood vessel sections on histology (%) (NS): 43.5 vs. 48.3 vs. 51.8.
Hom et al., 1992 [190]	ECGF ± heparin	Rabbit; ear island flap	46 (day-2 ligation:24, day-3 ligation: 26)	Topical (Gelfoam); ECGF 1800 µg/mL ± heparin 7 µg/mL	Saline	-Flap viability (%): day-2 ligation ** 26.7 vs. 11.1; day-3 ligation ** 44.3 vs. 20.9 -Vascularity (%): day-2 ligation * 10.9 vs. 7.3; day-3 ligation (NS) 12.2 vs. 9.7
Liu et al., 2022 [191]	SDF-1α (in microcarrier)	Mouse; caudal-based dorsal random flap (1.5 × 4 cm)	12 (3/group)	Implant (wound bed); IO; 2 mg microcarriers (SDF-1α 2.86 µg/mL)	NoTx; MC; MC@Mat	-Survival area (%) at POD7 ***: MC@SDF-1@Mat 80.0 vs. MC@SDF-1 53.5 vs. MC@Mat 31.2 vs. MC 18.8 vs. NoTx 15.1 -CD31+ vessel density **: higher in MC@SDF-1@Mat vs. other groups
Carroll et al., 1998 [192]	rhPDGF-BB	Hairless mouse; LD thoracodorsal island muscle flap (2 × 1.2 cm)	40 (10/group)	IM (LD muscle); D−10; 500 µg in 0.5 mL (1 mg/mL), divided prox/mid/dist	NoTx; Veh; Delay (10 d bipedicled)	-Survival area (%) **: PDGF 100 vs. Delay 73 vs. NoTx 37 vs. Veh 37 -mid capillary/muscle fiber ratio **: PDGF 1.60 vs. Delay 0.53

* *p* < 0.05; ** *p* < 0.001; *** *p* < 0.0001; aFGF, acidic fibroblast growth factor (FGF-1); bFGF, basic fibroblast growth factor (FGF-2); d, day(s); Delay, vascular delay; dist, distal; D−X, X days before flap elevation; ECGF, endothelial cell growth factor; FGF, fibroblast growth factor; IA, intra-arterial; IM, intramuscular; inj., injection; IntraFascia, intrafascial injection; IO, intraoperative (immediately after flap elevation); LD, latissimus dorsi; Mat, Matrigel; MC, microcarrier(s); MC@Mat, Matrigel-coated microcarriers; MC@SDF-1@Mat, SDF-1α-loaded Matrigel-coated microcarriers; mid, middle; NoTx, no treatment; NS, not significant; PDGF-BB, platelet-derived growth factor-BB; POD, postoperative day; prox, proximal; r, recombinant; RB, recipient bed; rh, recombinant human; SC, subcutaneous (includes subdermal); SDF-1α, stromal cell-derived factor-1 alpha; Veh, vehicle; VEGF, vascular endothelial growth factor.

**Table 2:** Gene/Nucleic Acid-Based Delivery of Angiogenic Factors in Preclinical In Vivo Flap Models: Representative Studies.

Study	Vector/Cargo	Model	Delivery Route, Timing, Dose	Control	Follow Up	Key Outcomes
Zacchigna et al., 2005 [87]	AAV-VEGF165	Rat; epigastric cutaneous flap (5 × 8 cm)	SC; IO vs. D−7 vs. D−14; 1.5 × 10^11^ vp/150 µL	AAV-LacZ; saline	POD7	Necrotic area ↓: 23.0% (IO), 40.0% (D−7), 41.7% (D−14)
	AAV-VEGF165	Rat; TRAM musculocutaneous flap (5 × 8 cm)	IM; IO vs. D−7 vs. D−14; 1.5 × 10^11^ vp/150 µL	AAV-LacZ; saline	POD7	Necrotic area ↓: 38.1% (D−7), 50.0% (D−14); no benefit at IO
Wang et al., 2011 [88]	AAV2-VEGF	Rat; McFarlane dorsal flap (3 × 10 cm)	ID; D−14; 3 × 10^10^ vp total (0.1 mL × 21; total 2.1 mL)	AAV2-GFP; saline	POD7	Viability ↑: 55.8 ± 6.9% vs. 45.9 ± 6.3% * (GFP) vs. 48.7 ± 4.9% ** (saline); vascularization/VEGF ↑
Huang et al., 2006 [193]	Ad-VEGF165	Rat; dorsal skin flap (3 × 9 cm)	SC; D−7 vs. D−7 + D−14; 5 × 10^8^ PFU/0.5 mL	Ad.null; PBS	POD7	Viability %: D−7 + D−14 75 ± 2; D−7 71 ± 1; Ad.null 57 ± 2; PBS 54 ± 1
Lubiatowski et al., 2002 [78]	Ad-VEGF	Rat; epigastric skin flap (8 × 8 cm)	SC (ischemic area vs. midline); D−2; 10^8^ PFU/0.3 mL	Ad-GFP; No inj.	POD7/14	Necrotic + hypoxic zone % (POD7/14): No inj. 25.6/23.0; local 18.1/9.7 *; midline 19.8/11.8 *
Basu et al., 2014 [89]	pVEGF165 + MEA-ET	Rat; McFarlane dorsal flap (8 × 3 cm)	ID; POD0/2/4; 2 or 4 sites; 1 mg/mL + MEA-ET	NoTx; pVEGF only	POD7/14	Timing effect: POD0 or POD2 effective (POD4 NS). Survival %: POD7 92.2 ± 9 vs. 77.0 ± 8.5; POD14 86.7 ± 1 vs. 68.5 ± 7.8
Chang et al., 2021 [92]	pCA5-HIF-1α	Rat; modified McFarlane dorsal flap (8 × 1.6 cm)	ID; D−7; 6 sites; 1 µg/µL × 50 µL	Sham plasmid	POD1/7/14	Necrotic area ↓ (POD1/7/14); CD31+ vessel density ↑ (POD14)
Lubiatowski et al., 2002 [91]	Ad-VEGF; Ad-Ang1; combo	Rat; cremaster muscle tube flap	IA (ext iliac a., closed circuit); IO; 3.3 × 10^7^ PFU/0.1 mL	PBS; Ad-GFP	POD3/7/14	FCD ↑ (POD7/14) in Ad-VEGF/Ad-Ang1/combo; permeability: Ad-Ang1 lower early; Ad-VEGF highest at POD7
Rah et al., 2014 [84]	Ad-HGF	Rat; caudal musculocutaneous flap (3 × 9 cm)	SC; D−2 + POD0; 10^7^ PFU/800 µL × 8 sites	rhHGF; PBS	POD10	Survival %: 71.4 ± 5.9 (Ad-HGF) vs. 63.8 ± 6.4 * (rhHGF) vs. 39.2 ± 13.0 * (PBS)
Jafari et al., 2017 [194]	pCik-hHGF + EP	Rat; modified McFarlane dorsal flap (9 × 3 cm)	ID + EP; D−1 (24 h); 25 µg/25 µL × 4 sites	NoTx	POD7	Necrosis % ↓ (27.14 ± 7.46 vs. 35.23 ± 3.90) *; Laser index ↑; CD31+ vessel density ↑

* *p* < 0.05; ** *p* < 0.01; ↑ increase; ↓ decrease; a., artery; AAV, adeno-associated virus; AAV2, adeno-associated virus serotype 2; Ad, adenovirus; Ad.null, null (empty) adenoviral vector; Ang1, angiopoietin-1; CA5-HIF-1α, constitutively active hypoxia-inducible factor-1α (HIF-1α) construct; CD31, cluster of differentiation 31 (PECAM-1); D−X, X days before operation; EP, electroporation; ET, electrotransfer; ext, external; FCD, functional capillary density; GFP, green fluorescent protein; HGF, hepatocyte growth factor; hHGF, human HGF; IA, intra-arterial; ID, intradermal; IM, intramuscular; IO, intraoperative; LacZ, β-galactosidase gene; MEA, multielectrode array; No inj., no injection; NoTx, no treatment; NS, not significant; p, plasmid; PBS, phosphate-buffered saline; PFU, plaque-forming units; POD, postoperative day; rhHGF, recombinant human HGF; SC, subcutaneous (includes subdermal); TRAM, transverse rectus abdominis myocutaneous; VEGF, vascular endothelial growth factor; VEGF165, VEGF-A165 isoform; vp, viral particles.

**Table 3:** Cell-Based Therapies in Preclinical In Vivo Flap Models: Representative Studies.

Study	Cell Source	Model	Delivery Route, Timing, Dose	Control	Follow Up	Key Outcomes
Chehelcheraghi et al., 2020 [195]	BM-MSCs ± CEE	Rat; dorsal random skin flap (30 × 80 mm)	SC; IO; BM-MSCs 6 × 10^9^/0.5 mL × 12 sites; CEE 0.5 mL × 4 sites	NoTx; Veh	POD7	Viability & vessel counts ↑ ** (BM-MSC, CEE, combo vs. control); Viability & mast cells: BM-MSC+CEE > BM-MSC alone
Ding et al., 2020 [196]	BM-MSCs	Rat; dorsal three-territory perforator skin flap (10 × 2.5 cm)	IM (panniculus carnosus) at choke zone II; IO; 10^5^ or 10^6^	PBS	POD7	Survival area ↑ *: High-dose BM-MSCs > low-dose BM-MSCs or PBS; microvessel diameter ↑ (25.32 µm vs. 13.07 µm (PBS))
Tang et al., 2016 [197]	BM-MSCs	C57BL/6 mouse; inferior epigastric cutaneous flap with I/R (3.5 h ischemia)	IV (femoral v.); after 1 h from reperfusion; 3 × 10^6^/150 µL	PBS (I/R control); sham	POD3, 5, 7, 14	Necrosis ↓ * POD3,5,7 (POD 3 (7.8% vs. 22.5%)); all flaps survived by POD14.
Leng et al., 2017 [198]	hUC-MSCs transfected with “F-5” gene	Rat; abdominal perforator skin flap (3 × 6 cm) with I/R (6 h ischemia)	SC; IO; 4 × 10^4^/0.1 mL × 10 sites	Saline	POD7	Necrotic area: 2% (F-5–hUC-MSC) vs. 39% (vector) vs. 41% (hUC-MSC) vs. 100% (saline).
Foroglou et al., 2019 [199]	Autologous GFP-ADSCs	Rat; bilateral dorsal random-pattern skin flaps (2 × 8 cm)	ID; IO; 10^6^/mL	Veh (PBS)	POD7	Necrosis ↓ *: 6.9 (4.2) → 3.1 (2.8) cm^2^ and 43 (26) → 19 (18)% (control → ADSC); endothelial differentiation suggested by fluorescence/IHC
Feng et al., 2020 [200]	hADSCs	Nude mouse; unipedicled SIEA axial flap with random extension (3 × 3 cm)	IA (Rt. femoral a.); IO; 10^3^,10^4^,10^5^/0.2 mL	IA PBS; Sham: no vessel ligation	POD7	Necrosis ↓, greatest at 10^4^ ** (20.71% vs. 52.62%); capillary density ↑ (e.g., 6.58 vs. 3.67)
Toyserkani et al. [201]	hSVF vs. hADSC ± hSVF lysate; autologous rSVF series	Rat; modified McFarlane distally based flap (2 × 7 cm + triangular area; 15 cm^2^), tubed	SC; IO; 5 × 10^6^/0.3 mL (0.1 mL/site). Lysate: 3 × 10^6^-cell equivalent	Veh (PBS)	POD7	Flap survival ↑ * with SVF (hSVF 55.7% vs. vehicle 45.3%); hADSC and SVF lysate: NS for survival; CD31 vessel density ↑ with hSVF & hASC
Zhang et al., 2018 [202]	hSVF-gel vs. SVF cell suspension	Nude mouse; dorsal random-pattern skin flap (1.5 × 4.5 cm)	RB deep fascia inj.; IO; 0.05 mL SVF-gel × 8 sites; ~1.53 × 10^6^/0.4 mL SVF cells in suspension	Veh (PBS)	POD14	Necrosis ↓ * with SVF-gel (22.1%) vs. SVF (35.5%) and PBS (53.8); CD31+ vessels ↑ (~43% vs. PBS); VEGF/bFGF expression ↑
Dong et al., 2022 [203]	Autologous SVF	Rat; pedicled abdominal fascial flap (2 × 5 cm, TDA pedicle) + free FTSG (2 cm circle)	IntraFascia inj.; IO; 4 × 10^6^	NoTx; Veh	POD1,2,3,7,10	Perfusion & survival ↑ (POD10 survival * 82.63% vs. 69.23%); CD31+ microvessels ↑
Jin et al., 2019 [204]	Allogeneic EPCs	Rabbit; abdominal superficial epigastric venous flap (10 × 6 cm)	SC; POD1; 10^5^/4 mL	Veh (PBS); sham (a. preserved)	POD10	Survival ↑ at POD10 * (58.4% vs. 4.8%); perfusion ↑ at POD10 * (157.4 vs. 103.9 PU); VEGF & eNOS ↑

* *p* < 0.05; ** *p* < 0.001; ↑ increase; ↓ decrease; a., artery; ADSC, adipose-derived stem/stromal cell; ASC, adipose-derived stem cell; bFGF, basic fibroblast growth factor; BM-MSC, bone marrow-derived mesenchymal stromal/stem cell; CD31, cluster of differentiation 31; CEE, chicken embryo extract; EPC, endothelial progenitor cell; FTSG, full-thickness skin graft; GFP, green fluorescent protein; hADSC, human ADSC; hSVF, human SVF; hUC-MSC, human UC-MSC; IA, intra-arterial; ID, intradermal; IHC, immunohistochemistry; IM, intramuscular; IntraFascia, intrafascial injection; I/R, ischemia–reperfusion; IO, intraoperative; IV, intravenous; MSC, mesenchymal stromal/stem cell; NoTx, no treatment; NS, not significant; PBS, phosphate-buffered saline; POD, postoperative day; RB, recipient bed; Rt, right; SC, subcutaneous (includes subdermal); SIEA, superficial inferior epigastric artery; SVF, stromal vascular fraction; SVF-gel, stromal vascular fraction gel; TDA, thoracodorsal artery; UC-MSC, umbilical cord-derived MSC; Veh, vehicle; VEGF, vascular endothelial growth factor.

**Table 5:** Pharmacologic and Small-Molecule Modulators in Preclinical In Vivo Flap Models: Representative Studies.

Study	Modulator (Class, Target	Model	Delivery Route, Timing, Dose	Control	Follow Up	Key Outcomes
Yao et al., 2024 [118]	Biliverdin (antioxidant/cytoprotective)	Mouse; dorsal random-pattern skin flap (1.2 × 3.6 cm)	SC; POD0,2,4,6; 5 mg/kg	Saline control; NAC 5 mg/kg	POD7	Survival & perfusion ↑ (Laser Doppler) at POD7; angiogenesis ↑ (α-SMA+ vessels, VEGF); oxidative stress/inflammation ↓ (iNOS↓, eNOS/HO-1/SOD-1 ↑); apoptosis ↓ (TUNEL, Bax ↓; Bcl-2 ↑); mechanism consistent with PI3K/Akt-mediated Nrf2 activation
Shafighi et al., 2011 [94]	DMOG (PHD inhibitor)	Rat; caudally pedicled modified McFarlane dorsal random-pattern skin flap (3 × 9 cm)	IP; D-3, IO, POD2; 40 mg/kg	IP saline	POD7	Distal necrosis ↓ (35.95% vs. 44.42%) *; Laser Doppler perfusion ↑ in the proximal two-thirds
Sergesketter et al., 2019 [119]	DMOG (PHD inhibitor)	Rat; McFarlane dorsal pedicle skin flap (3 × 6 cm)	IP ± topical; D-7 to POD7 (daily); 6/12/24/48 mg/kg/day (IP + topical) or 48 mg/kg/day (topical-only)	IP PBS/topical DMSO (veh)	POD3,7	Necrosis ↓ & perfusion ↑ (dose-dependent; POD3/7); HIF-1α staining ↑ with CD31+ vessels/VEGF ↑; TUNEL+ apoptosis ↓; systemic toxicity signals ↔.
Zeng et al., 2024 [68]	DMOG (PHD inhibitor)	Rat; multi-territory dorsal perforator flap (12 × 3 cm)	IP; POD1–3; 40 mg/kg	Saline/YC-1(HIF-1α inhibitor) 10 mg/kg	POD7	DMOG: Viability ↑ & Choke II MVD ↑ with HIF-1α/VEGF ↑; YC-1: reciprocal pattern (↓ viability, ↓ MVD, ↓ HIF-1α/VEGF).
Tao et al., 2023 [214]	FG-4592 (roxadustat; PHD inhibitor)	Rat; DCIA-based dorsal island multi-territory perforator flap (2.5 × 10 cm)	PO; QOD (D-7 to pre-op 2 h); 60 mg/kg/day	Tap water	12 h, POD7	Survival ↑ POD7 (89.40% vs. 82.61%) *; Choke II perfusion ↑ at 12 h and POD7 *
Zhang et al., 2021 [215]	SEW2871 (S1PR1 agonist)	Rat; dorsal random-pattern skin flap (8 × 3 cm)	IP; D0-POD7; 0.5 mg/kg/day	Untreated flap; DMSO (veh)	POD7	Survival ↑ (~30% vs. DMSO/untreated) *; angiogenesis ↑ (CD31 MVD; VEGFA/bFGF ↑); apoptosis ↓ (TUNEL; cleaved caspase-3/PARP ↓)
Lin et al., 2019 [125]	Pravastatin (statin)	Rat; modified McFarlane caudal random-pattern skin flap (3 × 9 cm)	IP; D0-POD7; 2 mg/kg/day	IP saline	POD7	Survival ↑ (69.44% vs. 54.80%) with edema ↓ (water content 44.07% vs. 63.89%) * and perfusion ↑ (blood-flow area 67.75% vs. 32.51%) *
Ye et al., 2023 [123]	Rosuvastatin (statin)	Mouse; dorsal random-pattern skin flap (4.5 × 1.5 cm)	IP; D0–POD7; 10 mg/kg/day; +3-MA (15 mg/kg/day IP) or +Compound C (1.5 mg/kg/day IP), given ~30 min pre-dose	IP saline	POD3,7	Survival ↑, edema ↓, LDBF perfusion ↑ with angiogenesis ↑ (CD34+ microvessels; VEGF/Cadherin5/MMP9 ↑). Autophagy ↑ (LC3II/Beclin1/CTSD ↑, p62 ↓); 3-MA or Compound C → benefit ↓/abolished.
Jia et al., 2017 [124]	Atorvastatin (statin)	Rat(diabetic); caudally based McFarlane-type dorsal skin flaps (3 × 10 cm)	PO; D-14 -POD7; 10 mg/kg/day	Veh gavage (0.5% methylcellulose)	POD7	Necrosis ↓/survival ↑ with capillary density ↑ (CD31+); circulating EPCs ↑ and EPC recruitment to flap ↑ (CD34+/Flk-1+); diabetic EPC migration ↑ and tube formation ↑.
Cui et al., 2019 [127]	Dietary nitrate (NaNO_3_; NO donor)	Rat; modified McFarlane caudal-based random-pattern skin/panniculus carnosus flap (3 × 9 cm)	PO; D-7 -POD7;5 mmol/L	DW/NaCl 5 mmol/L	POD1,3,7	Survival ↑ (POD3/7) with distal perfusion ↑ (LDF at 5 cm) and CD34^+^ microvessel density ↑; serum nitrate/nitrite ↑ and TNF-α/IL-6 ↓.
Tang et al., 2023 [126]	CN-Patch (CO + NO dual-gas hydrogel)	Rat; DCIA pedicled island perforator flap (11 × 3 cm) with I/R (5 h ischemia) + jugular AVF patency model	Topical, adventitial; IO; CORM-3 200 μM + GSNO 400 μM/500 μL per choke zone	Control/blank patch; CORM + GSNO inj.; delay	Intra-op 15–30 min, POD1,7,14,28,90	Early perfusion ↑ (laser speckle; 7.4× vs. CORM + GSNO) and survival area ↑ (+33.5% vs. control) with CD31/VEGF ↑ and IL-6/TNF-α ↓; AVF patency ↑ with intimal hyperplasia ↓ (neointima/lumen 0.132 vs. 0.224).

* *p* < 0.05; ↑ increase; ↓ decrease; D−x, x days before surgery; D0, day of surgery; PODx, postoperative day x; QOD, every other day; ↑, increase; ↓, decrease; ↔, no meaningful change; 3-MA (3MA), 3-methyladenine; Akt, protein kinase B; AVF, arteriovenous fistula; Bax, BCL2-associated X protein; Bcl-2, B-cell lymphoma 2; CD31, cluster of differentiation 31 (PECAM-1); CD34, cluster of differentiation 34; CM, conditioned medium; CO, carbon monoxide; CORM-3, carbon monoxide-releasing molecule-3; CTSD, cathepsin D; DCIA, deep circumflex iliac artery; DMOG, dimethyloxalylglycine; DMSO, dimethyl sulfoxide; DW, distilled water; eNOS, endothelial nitric oxide synthase; EPC, endothelial progenitor cell(s); Flk-1, fetal liver kinase-1 (VEGFR-2/KDR); GSNO, S-nitrosoglutathione; HIF-1α, hypoxia-inducible factor-1 alpha; HO-1, heme oxygenase-1; IL-6, interleukin-6; iNOS, inducible nitric oxide synthase; IO, intraoperative; IP, intraperitoneal; LC3II, microtubule-associated protein 1 light chain 3-II; LDBF, laser Doppler blood flow; LDF, laser Doppler flowmetry/imaging; MMP9, matrix metalloproteinase-9; MVD, microvessel density; NAC, N-acetylcysteine; NaCl, sodium chloride; NaNO_3_, sodium nitrate; NO, nitric oxide; p62, sequestosome 1 (SQSTM1); PBS, phosphate-buffered saline; PHD, prolyl hydroxylase domain; PO, oral (per os); SC, subcutaneous; TNF-α, tumor necrosis factor-alpha; TUNEL, terminal deoxynucleotidyl transferase dUTP nick-end labeling; VEGF, vascular endothelial growth factor; YC-1, HIF-1 inhibitor; α-SMA, alpha-smooth muscle actin.

## Data Availability

The data presented in this study are available on request from the corresponding author.
